# Stimuli-Responsive Polymer Actuator for Soft Robotics

**DOI:** 10.3390/polym16182660

**Published:** 2024-09-21

**Authors:** Seewoo Kim, Sang-Nam Lee, Ambrose Ashwin Melvin, Jeong-Woo Choi

**Affiliations:** 1 Department of Chemical and Biomolecular Engineering, Sogang University, 35 Baekbeom-ro, Mapo-gu, Seoul 04107, Republic of Korea; swkim@sogang.ac.kr; 2 Uniance Gene Inc., 273, Digital-ro, Guro-gu, Seoul 08381, Republic of Korea; snlee9191@hanmail.net

**Keywords:** polymers, sensor, actuator, external stimuli, soft biohybrid robotics

## Abstract

Polymer actuators are promising, as they are widely used in various fields, such as sensors and soft robotics, for their unique properties, such as their ability to form high-quality films, sensitivity, and flexibility. In recent years, advances in structural and fabrication processes have significantly improved the reliability of polymer sensing-based actuators. Polymer actuators have attracted considerable attention for use in artificial or biohybrid systems, as they have the potential to operate under diverse conditions with high durability. This review briefly describes different types of polymer actuators and provides an understanding of their working mechanisms. It focuses on actuation modes controlled by diverse or multiple stimuli. Furthermore, it discusses the fabrication processes of polymer actuators; the fabrication process is an important consideration in the development of high-quality actuators with sensing properties for a wide range of applications in soft robotics. Additionally, the high potential of polymer actuators for use in sensing technology is examined, and the latest developments in the field of polymer actuators, such as the development of biohybrid polymers and the use of polymer actuators in 4D printing, are briefly described.

## 1. Introduction

There has been a significant increase in the number of polymer materials of various types that have been developed, and they have been designed for specific uses by considering their physical and chemical properties. These materials have a remarkable ability: their properties can be varied in real time by employing a wide range of external triggers, such as electricity, magnetism, pH, and light [1]. Polymers with properties that change in response to external stimuli have become an essential component of smart materials [2]. Their versatility is impressive, as they can have a variety of forms; in other words, they can be a solid, a liquid, a gel, or a 2D thin film, and they can comprise nano- or microparticles. Their quick response to internal and external stimuli has not only contributed significantly to the development of smart materials but has also led to them being used for purposes such as the development of sensors [3,4,5] with fast response times, the controlled and timely release of drugs and other sensitive reagents, detection, and the fabrication of self-healing materials. The remarkable properties of polymers make them a potential candidate for use in the fabrication of sensors [6]. Their high stability and the development of facile fabrication techniques have led to them being indispensable in the field of sensor fabrication [7,8,9].

**Figure 1:**
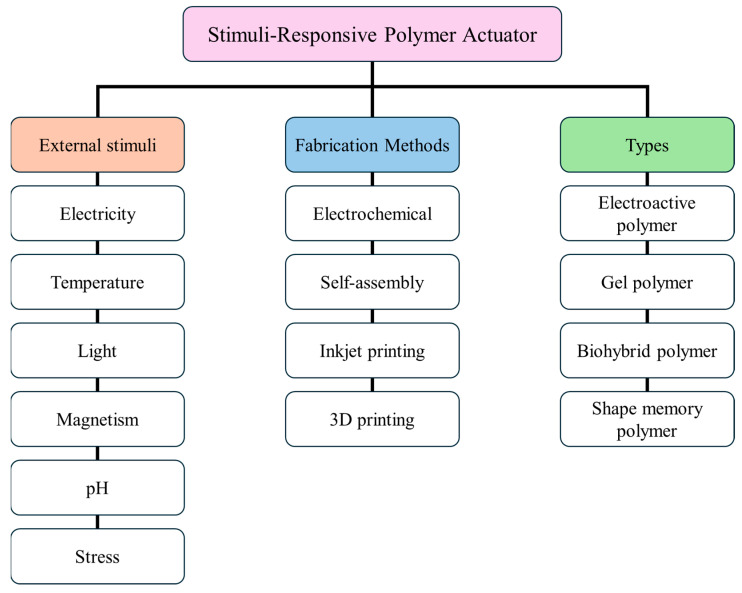
Schematic classification of stimuli-responsive polymer actuators by types of external stimuli, fabrication methods, and types.

The field of smart, flexible polymer actuators has witnessed significant developments in recent years owing to advances in fabrication techniques [10,11]. These actuators have significantly influenced the sensor industry, as their use has increased considerably. Electrochemical, self-assembly, dispersion, and 3D printing methods have all played a pivotal role in the development of effective and stable actuators, which have now become indispensable. Their exceptional flexibility [4] makes them a fascinating research subject with potential applications in various fields. Their potential opens new possibilities for their use in sensing and actuation, and researchers continue to explore their potential in many applications and fields. The most promising applications of polymer actuators are in biomedical and health monitoring devices, smart soft robotic skin, wearable and flexible actuators in electronics, and, most importantly, flexible sensors [12,13,14,15,16]. These sensors are designed to detect and confirm body movements, and they are becoming an essential component of modern sensor technology. The field has been significantly influenced by the use of polymer actuators, especially polyaniline (PANI) [17] and poly(3,4-ethylenedioxythiophene):polystyrene sulfonate (PEDOT:PSS) [18]. By exploiting the distinctive characteristics of these polymers, researchers have made significant progress in developing sophisticated devices that can be seamlessly integrated into our daily routines. This comprehensive review delves into the fascinating world of polymer actuation and the potential use of polymer actuators as sensors. It provides an in-depth analysis of various polymers that exhibit actuation properties, describes their unique properties, and discusses how they can be harnessed as sensors. The review also highlights the different stimuli (refer to Figure 1) that can trigger the actuation response of these polymers and discusses how sensitive the polymers are to the stimuli. Furthermore, it describes various fabrication techniques of polymer actuators and mentions their advantages and disadvantages. Additionally, the review discusses the applications of actuators.

## 2. Types of Polymer Actuators

Polymer actuators are advanced materials that have many advantages over traditional metallic and ceramic actuators. They are lightweight, flexible, and cost effective. They can flex, bend, or expand in response to various triggers, such as changes in temperature, exposure to electricity, variations in light intensity, the application of a magnetic field, and changes in the pH level. Polymer actuators are important components in various devices and systems, including medical devices, prostheses, robots, toys, biomimetic devices, and micro/nanoelectromechanical systems. These advancements include the use of gels that can be triggered by external stimuli, pioneering active stimuli mechanisms in polymers as a result, which empower them to execute intricate movements that show shrinkage [19], stretching [20], twisting [21,22], curling [23], swimming [21], crawling [22], and circular motions [24]. Common examples of polymer actuators include electroactive polymers, gel actuators, shape memory polymers, and biohybrid polymers.

### 2.1. Electroactive Polymer

**Figure 2:**
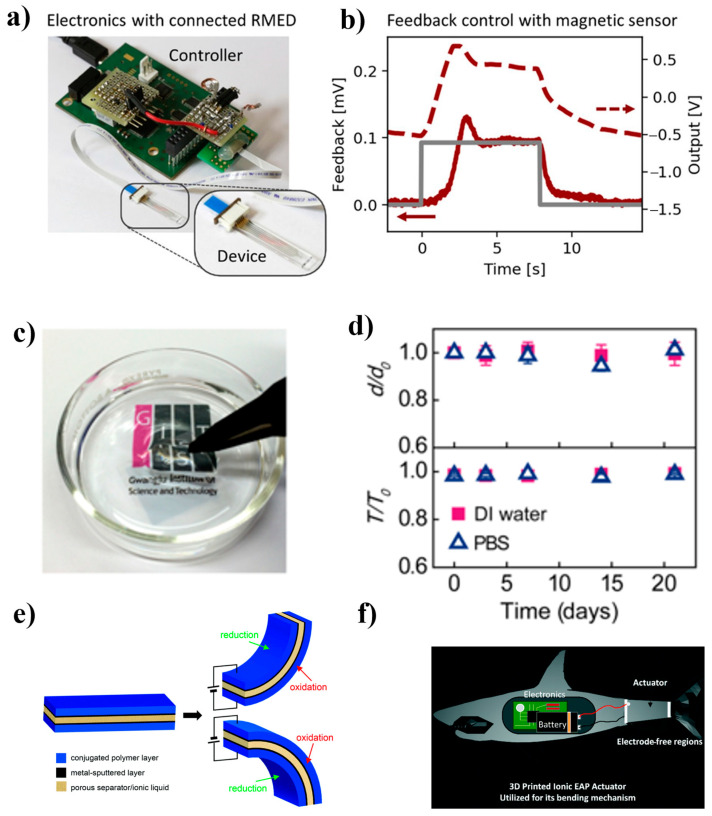
(**a**) Operation model of custom-developed microelectronics devices for device interfacing and experimental controlling. (**b**) Setpoint, feedback, and output curves of a typical feedback-driven positioning experiment. Position feedback was provided by integrated magnetic sensors. The proportional gain constant was set to *K*_P_ = 200. Reprinted with permission from reference [29]. (**c**) A photograph of the c-PEDOT:PSS-PET substrate immersed in water. (**d**) Plots of the normalized thickness (upper) and optical transmittance of the c-PEDOT:PSS films (lower) concerning the duration of immersion in DI water and phosphate-buffered saline (PBS). Reprinted with permission from reference [30]. (**e**) Schematic illustration of the actuation mechanism in the case of a trilayer conjugated polymer. Reprinted with permission from reference [26]. (**f**) Prospective 3D printed soft robot, where both the EP actuator and robotic body are printed using a single printing process. Cut-away view to show the internal components. Reprinted with permission from reference [28].

Electroactive polymers (EPs) are at the forefront of material science, and they have a remarkable ability: their structure or size changes when an external electric field is applied. They can be one of two types, namely electronic and ionic EPs [25]. Both are conducting polymers, and along with ionic polymer–metal composite-based actuators, they are the most common examples of EPs. For instance, a polypyrrole-based trilayer bending actuator can a show simple bending motion in the presence of an externally applied electric field. The trilayer material flexes and deforms in response to the presence of an electrolyte in a poly(vinylidene fluoride) (PVDF) separator. Figure 2e shows the actuation mechanism of trilayer conjugated polymer actuators [26]. In shape-controlled devices, EP actuators are incorporated with electronic sensors at the microscale level; the sensors are used to regulate the actuators. The resulting microscale devices exhibit multifunctionality, and they can grasp, hold, and release biological tissues such as neuronal bundles. EPs can also be used as synthetic muscles, as reported by Ras Labs [27]. One such biological function of fish was demonstrated using EPs, as shown in Figure 2f. The fabrication involves a one-step process where the internal fittings, ionomeric polymer core, and electrode surface are printed in a single step [28]. One of the latest advances in robotics is the development of reshaped microelectronic devices. These innovative devices are capable of feedback-controlled actuation, as shown in Figure 2a, and this advancement is a significant milestone in the evolution of soft micro-robotics [29]. We used a specialized metal–organic sacrificial layer for the efficient and reliable release of ultrathin structures. These cutting-edge EP actuators showed rapid responses and were compatible with biological systems, which facilitated the dynamic reshaping of the device. Additionally, we devised a see-through conductive interface that could directly interact with cells. A high-performance, water-resistant film made using solvent-assisted crystallization to create PEDOT:PSS. Our films exhibited remarkable stability in water over extended periods without dissolving or disintegrating. Furthermore, crystallized PEDOT:PSS deposited on a polyethylene terephthalate substrate has been found to exhibit remarkable electrical conductivity and optical properties [30]. Overall, EPs have gained popularity because of their flexible and lightweight nature and relatively high tolerance to strain. Their high strain tolerance allows them to be compressed or molded. Despite their interesting properties, EPs have disadvantages; for example, a high voltage is required for actuation. This has an adverse effect on the actuation force. Furthermore, their fabrication is tedious and complex, especially in the case of thin films with an extended bilayer or trilayer. The lower stability and deterioration of SPs after repeated cyclic actuation is also a matter of concern.

### 2.2. Polymer Gel

A polymer gel is a solid-like material consisting of a three-dimensional crosslinked framework filled with fluid. Gels can respond to environmental changes such as changes in heat, electricity, light intensity, and pH level [31,32,33]. Thermally responsive hydrogels have been extensively researched, as they undergo a transition from hydrophilic to hydrophobic at a critical temperature called the lower critical solution temperature. The hydrophilic segments of hydrogels dissolve or swell in water at lower temperatures, while hydrophobic interactions cause the hydrogels to shrink at higher temperatures. Polymer gels have garnered considerable attention because of their promising potential for use in lithium metal batteries. Owing to their excellent electrochemical properties, especially in the context of wearables, and flexibility, they are seen as effective in addressing safety-related concerns.

**Figure 3:**
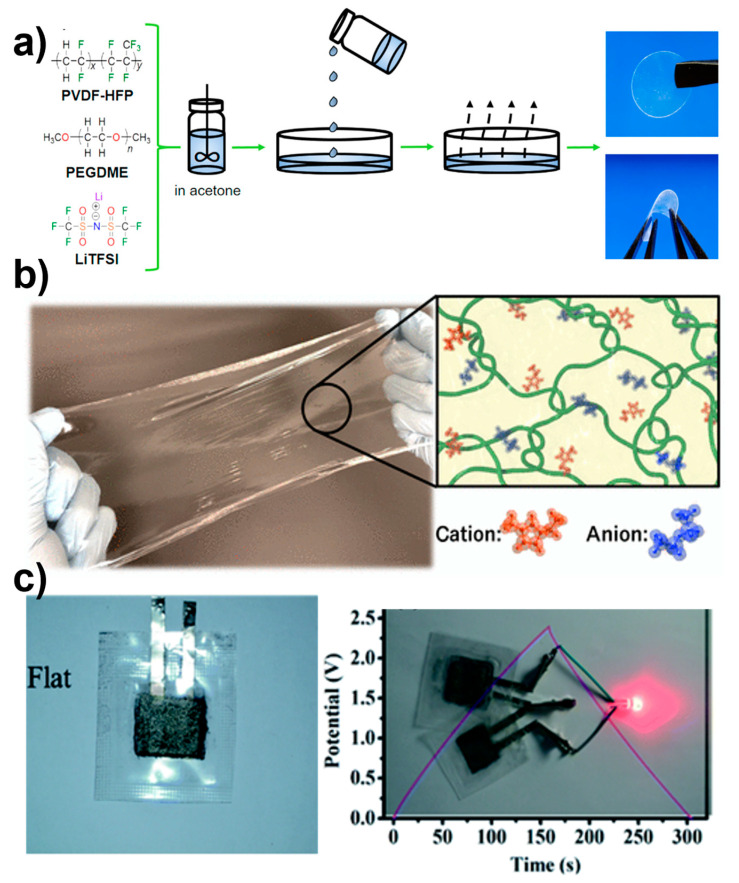
(**a**) Schematic illustration of the preparation process. Reprinted with permission from reference [35]. (**b**) Photograph and schematic illustration of the ultrahigh-molecular-weight gel [36]. (**c**) Photograph of a flat individual polyelectrolyte-based GPE (PGPE) and of an LED powered by a 2.4 V device; here, two 1.2 V PGPE supercapacitor devices are connected in series. Reprinted with permission from reference [34].

A straightforward and scalable technique for preparing a gel polymer electrolyte (GPE) involves the use of the very safe plasticizer polyethylene glycol dimethyl ether embedded in a polymer matrix of poly(vinylidene fluoride-co-hexafluoropropylene), as shown in Figure 3a. A new GPE that we developed has outstanding safety features. It is nonflammable and remains stable at high temperatures up to 250 °C. At room temperature, the material shows remarkable electrochemical properties, such as a high ionic conductivity of 3.4 × 10^−4^ S cm^−1^ and a high lithium transference number [24]. A highly stretchable and self-healing polymer gel has been formed through a straightforward one-step process involving physical entanglements of ultrahigh-molecular-weight polymers. This resulted in the formation of transparent polymer gels with excellent properties, including high stretchability, high ionic conductivity, and recyclability (Figure 3b) [25]. Similarly, a polyelectrolyte-based gel polymer electrolyte (PGPE) containing anions and cations has been fabricated with repeated units [34]. The presence of charged groups in the polyelectrolyte was exploited to enhance its mechanical strength and conductivity properties (0.99 MPa tensile strength and 66.8 mS cm^−1^ ionic conductivity). The presence of cations and anions between the charged groups generated a solid electrostatic force between the charged groups and water molecules, resulting in the facile separation and migration of the cations and anions. This led to the PGPE having high conductivity. Furthermore, the charged groups increased the adhesion between the electrode and PGPE, which lowered the interface impedance. Consequently, the PGPE exhibited high specific capacitance, high energy, and high power density. It was found that the supercapacitor’s specific capacitance and charge–discharge behavior did not change when it was bent. This allowed the PGPE device to power a red LED with the help of two 1.2 V supercapacitors when connected in series, as shown in Figure 3c.

### 2.3. Biohybrid Polymer

Recent progress in the preparation techniques of polymer nanomaterials and improvements in our understanding of biomolecular structures and functions have made it possible to develop advanced polymer-based biohybrid nanostructures (PBBNs). Polymerization-induced self-assembly (PISA) is an innovative and versatile approach for effectively producing high concentrations of polymeric nano-objects, and it has significant benefits over current self-assembly approaches. Consequently, it is being increasingly used to manufacture PBBNs [26].

**Figure 4:**
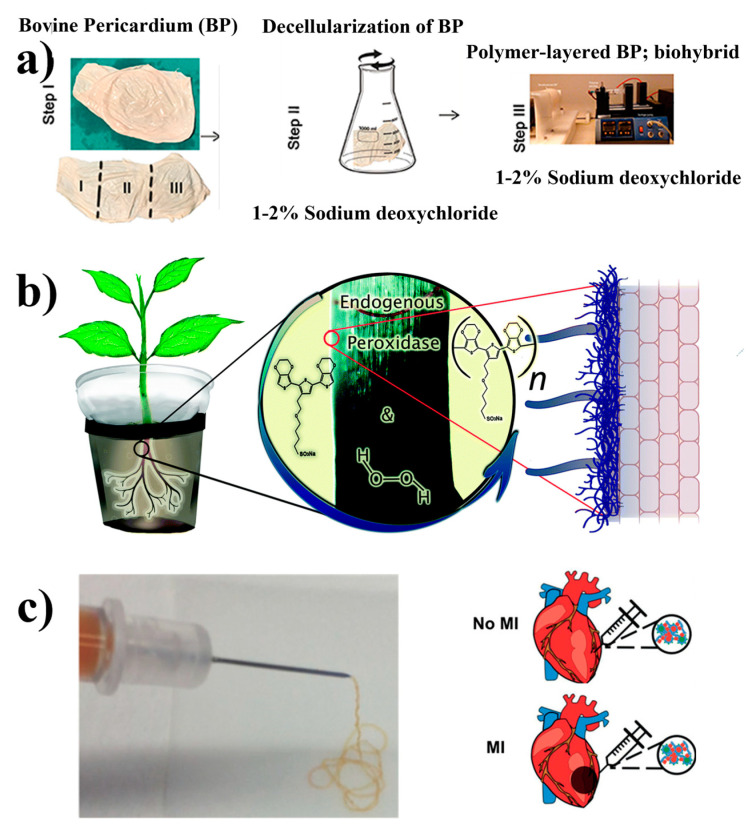
(**a**) The biohybrid composite fabrication steps are conducted using bovine pericardium (BP). Step I: intact BP is divided into three portions for processing, and the first portion is left untreated. Step II: the second portion of the sac is decellularized using sodium deoxycholate. Step III: coating of polycaprolactone: chitosan polymer layer on the decellularized BP via electrospinning. Reprinted with permission from reference [43]. (**b**) Electronic functionalization of plant roots. ETE-S polymerizes on the roots of intact bean plants catalyzed by endogenous plant cell wall peroxidases and H_2_O_2_. Reprinted with permission from reference [48]. (**c**) Ejection of guest–host granular hydrogel from the syringe through a 27 G needle onto a surface, and two-component granular hydrogels injected into rat hearts either with myocardial infarction (MI) or no MI. Reprinted with permission from reference [47].

A novel hybrid material, namely the combination of a decellularized pericardial extracellular matrix with polymeric nanofibers, is being investigated as an innovative substitute for cardiovascular tissue. Its production involves the removal of cells from pericardial sacs by using sodium deoxycholate and the subsequent application of polycaprolactone chitosan fibers onto the matrix through a technique called electrospinning, as depicted in Figure 2a. To understand the interaction between the tissue and the polymer, researchers have used spectroscopic methods to extensively investigate its mechanical properties. Initial findings indicate promising cellular adhesion and survival rates. A decellularized matrix encapsulated with a polymeric nanofiber coating exhibits favorable attributes that make it a compelling candidate for replacing cardiovascular tissue [27]. In an astounding leap forward, researchers have developed biohybrid plants that can be seamlessly integrated with a modified root system, as depicted in Figure 4b; introducing a solution of conjugated oligomers in the plants facilitated the polymerization of oligomers onto their root, and a widespread network of readily available conductors was formed. Remarkably, these plants exhibited no adverse effects upon being electronically modified and functionalized. Rather, they adapted to their newly modified hybrid system by developing an intricate root structure. This innovative approach holds great promise for use in a diverse array of biohybrid applications [37,38,39,40,41,42,43,44]. It also represents a significant advancement in the field of biofabrication, where biological organisms are used to fabricate functional materials and composites. Furthermore, this research has improved our understanding of the interaction between biological tissue and artificial materials, and it has paved the way for the seamless integration of electronics and biology, heralding the development of sophisticated communication pathways between these two domains [28]. Biohybrid polymers include an important class of gels known as hydrogels or microgels [45,46]. These have attracted considerable attention for their biocompatibility and nontoxic degradability, which render them suitable for use in tissue repair and other biomedical applications. Granular hydrogels with multifunctional properties have been synthesized using a model based on guest–host interactions [47]. The synthesis process involves the use of hyaluronic acid microgels and modular, molecular intraparticle covalent crosslinking. Granular hydrogels can be injected through needles as small as 27 G (~210 µm inner diameter), as shown in Figure 4c. Cyclodextrin and adamantane have been used for guest–host interparticle crosslinking. Injectable hydrogels have garnered interest for the treatment of myocardial infarction, as they can provide mechanical support or deliver therapeutics, as shown in Figure 4c.

### 2.4. Shape-Memory Polymers

**Figure 5:**
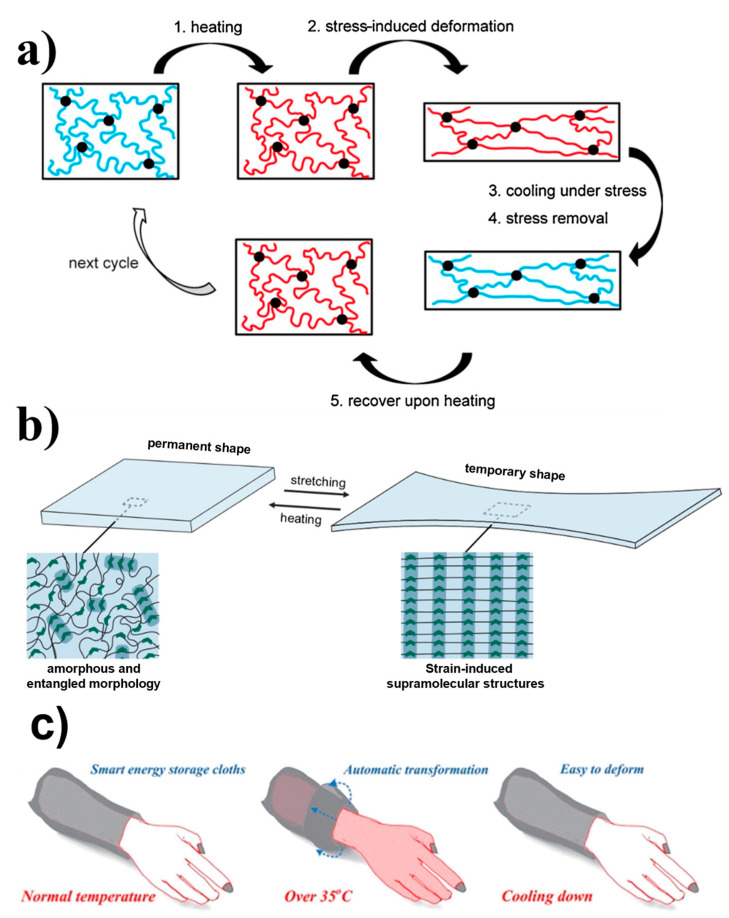
(**a**) The molecular mechanism of the dual-SME. Black dots: net points; blue lines: molecular chains of low mobility below *T*_trans_; red lines: molecular chains of high mobility above *T*_trans_. Reprinted with permission from reference [53]. (**b**) The alignment of polymer chains, i.e., entangled in their permanent form and aligned when stretched in their temporary form. Reprinted with permission from reference [52]. (**c**) Schematic demonstration of this smart shape-memory textile used in a smart cloth. Reprinted with permission from reference [55].

Shape-memory polymers (SMPs) are a fascinating class of polymers with a unique property: their shape changes upon exposure to external stresses. After deformation, they can maintain their new shape temporarily owing to the formation of reversible crosslinks [49,50,51,52,53,54]. A visual representation is provided in Figure 5a [29]. In the presence of external triggers, such as changes in temperature or irradiation with light, SMPs exhibit an extraordinary capability to return to their initial shape. These materials are lightweight and highly elongated, which make them well suited for use as organic actuators. Extensive research has been conducted on the thermally induced shape-memory effect (SME), which is an intriguing phenomenon in which a material returns to its original shape when subjected to temperature variations. The programming process involves heating, distorting, and cooling the sample. When the SMP is heated above a specific temperature, it triggers the SME, resulting in a return to the stored permanent shape [30]. When polymers are subjected to a strain, they can form supramolecular structures with high energy density. When a force is applied to the polymer, the molecular chains become aligned and form powerful directional dynamic bonds, as depicted in Figure 5b. These bonds facilitate the formation of stable supramolecular nanostructures, effectively maintaining the chains in a stretched state. When subjected to heat, the dynamic bonds within the material break, which causes the stretched chains to revert to their initial disordered state [31]. In a unique real-time application, the SME was exploited to develop an innovative device known as a shape-memory supercapacitor (SMSC) [55]. An energy storage shape memory textile woven with SMSCs and traditional fabrics can work similar to intelligent sleeves. Hence, remembering its predesigned shape can activate automatic cooling in times of overheating. This has been well demonstrated for a predesigned curl shape; a smart sleeve was shown to automatically self-curl, thereby promoting heat dissipation and initiating automatic cooling, in cases where the wearer performs physical exercises or has a high body temperature.

## 3. Types of Polymer-Sensing Actuators and the Influence of Stimuli

### 3.1. Electroactive Polymer-Sensing Actuators

**Figure 6:**
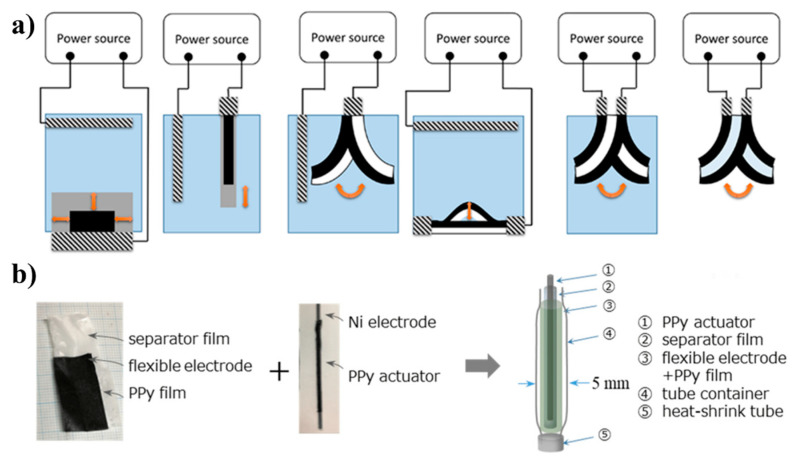
(**a**) Various actuator constructions using conducting polymers differ in bulk expansion, bending bilayer, buckling trilayer, and bending trilayer in air. Reprinted with permission from reference [56]. (**b**) Photograph and schematic illustration depicting the structure and assembly procedure of the actuator. Reprinted with permission from reference [57].

Electroactive actuators have garnered much more interest than conventional actuators because of their enhanced responsiveness to electrical stimuli. EPs can involve either of two primary mechanisms. One mechanism occurs in ionic EPs, where actuation is driven by ion diffusion between two coupled electrodes under a relatively low voltage (1–2 V). The second mechanism is found in field-activated EPs, where actuation occurs because of Coulombic forces under a relatively high electric field (>10 V/µm). Recently, conjugated polymers (CPs), which are characterized by high actuation strain, high compliance, low mass density, and ease of processing [32], have emerged as a unique class of polymers, with polypyrrole being a notable example, because of their inherently high conductivity. They have the potential to be used for the development of soft actuators or “artificial muscles” for a wide range of applications [33]. In recent years, our understanding of these materials and the mechanisms underlying their actuation has improved substantially. This progress can be attributed to the development of physical and electrochemical models. Currently, researchers are focusing on leveraging the advantages of CP actuators over other actuating materials. These advantages include their minimal energy demand and the possibility of miniaturizing them. Additionally, efforts are underway to overcome the intrinsic limitations of CP actuators. CP actuators are available in various forms, including films, filaments/yarns, and textiles, and they can operate in both liquids and air. Engineers can readily employ these actuators; they exhibit different actuation modes, as shown in Figure 6a [33]. Festin et al. developed interpenetrating polymer network (IPN) actuators by combining 3,4-ethylenedioxythiophene with a blend consisting of poly(ethylene oxide) and nitrile butadiene rubber. When these actuators were infused with ions from 1-ethyl-3-methylimidazolium bis(trifluoromethylsulfonyl)imide (EMITFSI) electrolyte, they could reach a saturation level of 134%. During testing, the researchers observed a 2.4% strain and applied a blocking force of 30 mN. The PEDOT-IPN actuator produced a voltage in the range of a few millivolts. The researchers predicted that the IPN actuators could serve as both actuators and sensors in various systems, including complex systems [34]. Palmre et al. developed a carbon–ionic liquid EP actuator (CIL-EPA) based on a carbon aerogel that changed its shape in response to electrical stimulation. Carbon aerogel is prepared by subjecting organic aerogel to pyrolysis, and it is valued for its large surface area and remarkably low density. The exceptional properties of this extraordinary material make it suitable for a diverse array of applications. Its porous structure and conductivity also make it ideal for use as an electrode. The researchers dried 5-methyl resorcinol-formaldehyde gel and pyrolyzed it in an inert gas atmosphere to obtain carbon aerogel. The CIL-EPA exhibited 5% higher graphitization compared with other carbide-derived, carbon-based actuators and a strain of 1.2% at a relatively low voltage of ±2 V [56]. Furthermore, a soft linear actuator was constructed and tested from polypyrrole, resulting in an easily synthesizable, cost-effective, and biocompatible EP. The resulting actuator, with a 5 mm diameter and resembling artificial muscles, contained a flexible counter electrode and could be operated in the air (see Figure 6b) [57].

### 3.2. Temperature-Sensing Polymer Actuators

**Figure 7:**
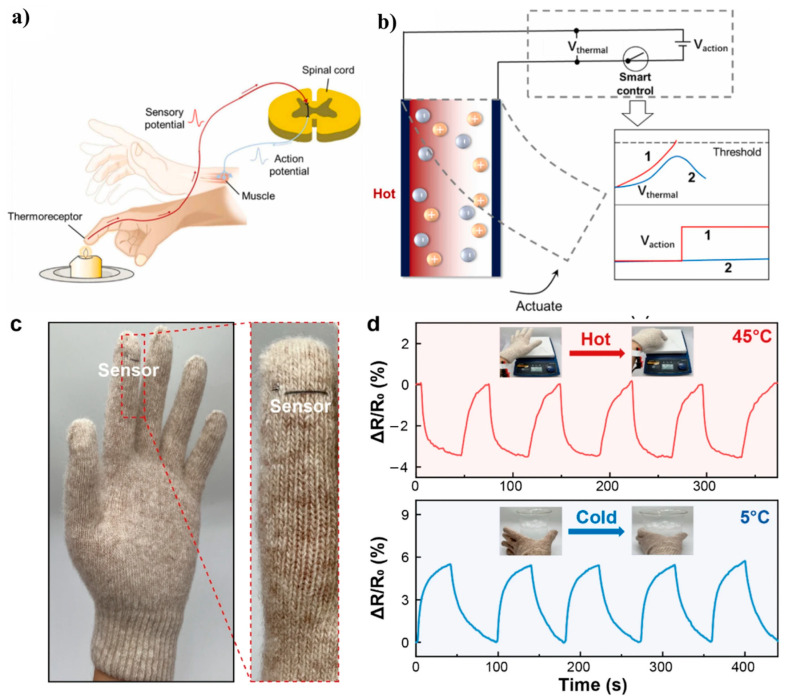
Thermal sensing actuator design principle. (**a**) Schematics showing the hand withdrawal reflex consists of a thermoreceptor, sensory neurons, spinal cord, motor neurons, and muscle. (**b**) Working mechanism of the TSA simulating the function of hand withdrawal reflex, with thermal sensing potential (V_thermal_), action potential (V_action_) and a smart control system. Reprinted with permission from reference [60]. (**c**) Image of the fiber temperature sensor sewn onto the tip of a hand glove. (**d**) Temperature response of the fiber sensor to repetitive touch of a hot (45 °C) or cold (5 °C) object. Reprinted with permission from reference [61].

An advanced thermal-sensing actuator (TSA) with dual functionality has been developed using conductive polymer ionogel electrodes. It can detect both temperature and radiation accurately. The cutting-edge design incorporates an advanced control system that effortlessly combines thermal sensing and actuation functions into a single device, facilitating precise and efficient operation (Figure 7a) [36]. Temperature measurement by the TSA is based on the variation of the elasticity modulus with temperature. For polymer optical fibers, the temperature can be accurately measured by monitoring changes in the refractive index resulting from stress variation in the polymer under a constant applied force. In the case of conducting polymers, the temperature is determined from changes in the electrical resistance. This approach involves the use of both positive temperature coefficient units, where the resistivity increases with the temperature, and negative temperature coefficient (NTC) units, where the resistivity increases as the temperature decreases [58,59]. The system allows for precise proprioceptive movement and provides valuable insights that can contribute to the improvement of autonomous robots. A fiber temperature sensor with a temperature-sensing core comprising a conductive polymer composite has been developed. The composite comprises thermoplastic polylactic acid, a conductive carbon filler, reduced graphene oxide, and a highly flexible, linear, low-density polyethylene passivation layer. This sensor exhibits remarkable sensitivity with a coefficient of −0.285%/°C in the 25–45 °C temperature range, and it boasts terse response and recovery times of 11.6 and 14.8 s, respectively. The fiber temperature sensor has been successfully integrated into everyday clothing, including a hand glove, as shown in Figure 7b [38]. The sensor consistently showed stable performance, effectively responding to changes in body temperature and accurately detecting temperature through touch. These findings highlight the sensor’s high potential for use in wearable technology, electronic skin systems, and biomedical devices.

Working mechanism of the TSA simulating the function of hand withdrawal reflex, with thermal sensing potential (V thermal), action potential (V action) and a smart control system. 

### 3.3. Optical-Sensing Polymer Actuators

Optical-sensing actuators integrate both sensing and actuation functions using light. These systems detect environmental changes on the basis of optical signals and produce a mechanical response or actuation. They typically involve materials and mechanisms that exhibit a change in their physical properties, such as their shape, volume, or refractive index, when exposed to specific wavelengths or intensities of light [38,39]. Ge et al. developed a photoresponsive actuator by combining semicrystalline poly(ethylene-co-vinyl acetate) (EVA) with gold nanoparticles (AuNPs). When a 532 nm wavelength laser was incident on the actuator, the heat generated by the AuNPs raised the temperature of the crystallite regions with a low melting temperature, leading to a contraction force that induced overall actuation. Once the laser was turned off, recrystallization occurred during cooling, and the expansion force restored the actuator to its original state. The EVA actuator showed a maximum angle change of 20°. When exposed to light with an intensity of 1.13 W/cm^2^, it showed a 60% strain and generated a contraction force of 0.7 N. The researchers suggested that an EVA actuator with a combination of different light-absorbing materials and heat-generating additives had high potential for use in various temperature-memory devices [40].

**Figure 8:**
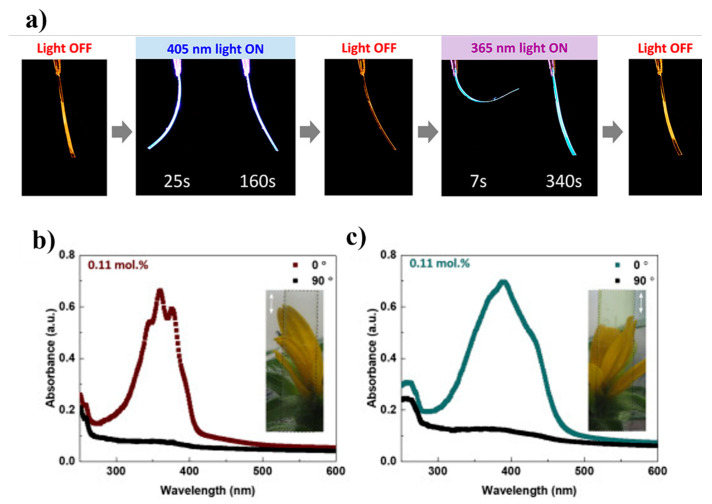
(**a**) Photoactuation of the polymer ribbons incorporating H1 [62]. Thickness 25 μm. (**b**,**c**) Linear dichroism of azobenzene-doped ultra-drawn ultrahigh-molecular-weight polyethylene films. Insets are photographs taken of the corresponding ultra-drawn films, with the transmission axis of the polarizer at 0° indicated in white. Reprinted with permission from reference [63].

Bistable hydrazones have been found to trigger (chiral) the deformation of polymer networks of liquid crystals, as illustrated in Figure 8a. The triggering mechanism involves the accumulation of photoinduced tension in the polymer, and the accumulated tension has minimal impact on the liquid crystalline order. Consequently, the incorporation of hydrazone-doped liquid crystal systems offers additional possibilities for light-responsive molecular actuators [41]. The actuators are fabricated using an ultra-drawn ultrahigh-molecular-weight polyethylene (UHMW-PE) material, and azobenzene photo-switches and polyethylene (PE) side chains are symmetrically attached to the structure. The extended PE side chains are intended to facilitate better dispersion within the nonpolar UHMW-PE matrix, resulting in the formation of highly aligned ultra-drawn films. In the presence of rotating linearly polarized light, the actuator exhibits a unique photoinduced stress wave response, as depicted in Figure 8b. This photo-mechanical response is characterized by rapid, high-stress, and low-strain behavior, which is atypical of soft polymer systems, and the actuator shows physical properties like those of hard metals and ceramics [42].

### 3.4. Magnetic-Sensing Polymer Actuators

Magnetic polymer composites are of interest because they are biocompatible, which implies that they can coexist with biological systems without harming them. Additionally, these composites have adjustable mechanical, chemical, and magnetic properties. Thus, these properties can be fine-tuned to suit specific applications. In particular, their manufacturing versatility facilitates the preparation of a wide range of products with diverse functionalities [43,44].

**Figure 9:**
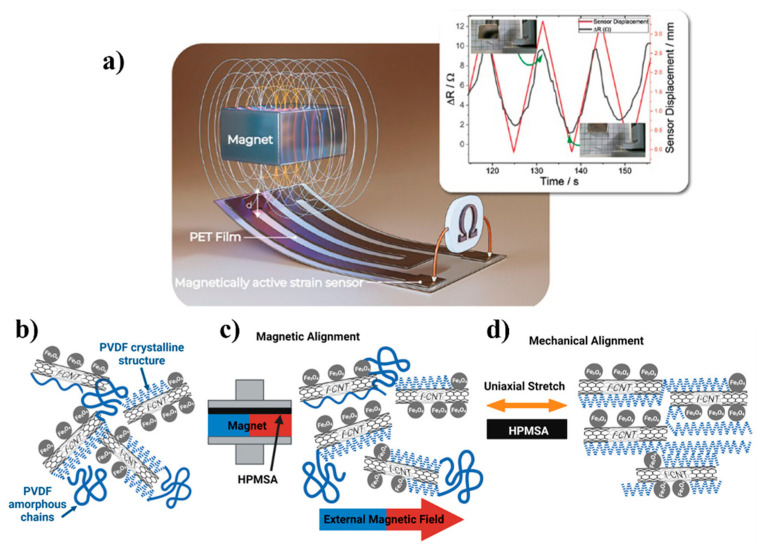
(**a**) Schematic representation of a magnetically active strain sensor on a PET film with resistance variation correlation with the magnetic actuator displacement after correction of the low-frequency offset. Inset resistance variation correlation with the magnetic actuator displacement after correction of the low-frequency offset. Reprinted with permission from reference [64]. Novel dual-alignment processing method for HPMSA to align magnetic particles and PVDF crystals. (**b**) Random Fe_3_O_4_, f-CNT, and amorphous and crystal phases of PVDF. (**c**) Magnetic alignment: movement of f-CNT and PVDF crystals due to Fe_3_O_4_ alignment with an external magnetic field. (**d**) Mechanical alignment: further alignment of Fe_3_O_4_, f-CNT, and PVDF crystals (transformation from amorphous phase) with mechanical uniaxial stretching of HPMSA [65].

A remarkable multifunctional polymer-based composite has been prepared with PVDF as the primary polymer matrix. It incorporates cobalt ferrite (CoFe_2_O_4_, hereafter abbreviated as CFO) and multi-walled carbon nanotubes (MWCNTs) as fillers, as shown in Figure 9a [45]. This composite shows magnetic and electrical characteristics, with a magnetization of 11.1 emu g^−1^. It has high potential for use in magnetic actuators owing to its ability to sense strain properties autonomously, and it exhibits excellent response and reproducibility. An innovative actuator, namely the hybrid piezoelectric–magnetic self-sensing actuator (HPMSA), has been developed to sense and actuate simultaneously. The film consists of iron oxide, functionalized carbon nanotubes, and polyvinylidene fluoride and is engineered to have high piezoelectric and magnetic characteristics; a dual-alignment method is used to fabricate it. In this process, solid elemental bonds are formed, leading to simultaneous alignment (see Figure 9b). Hence, the HPMSA can have 88% polar β-crystal content, significantly surpassing the 66% achieved through magnetic alignment alone. Operating over a range of frequencies, namely 40–600 Hz, the HPMSA serves as a vibration damper with a sensing sensitivity of 2.5 mV g^−1^ and a weighted accelerated damping of 0.72 ms^−2^, mitigating the risk to the health of passengers. The HPMSA holds considerable promise for enhancing safety. The future generation of transportation vehicles is expected to be characterized by their versatile design, ability to align simultaneously, and advanced vibration control features [46].

The HPMSA material is shown in Figure 8. In another similar study, during electrophoresis, carbon nanotubes were treated with PVDF, and hence, they were chemically bound. The presence of chemical bonds was confirmed through X-ray photoelectron spectroscopy and photoluminescence. This resulted in changes in the α, β, and γ of the polymer, which could be exploited, and significant changes occurred in the permittivity and conductivity of the material. This allowed for a new perspective to be studied to understand the mechanism underlying PVDF fiber inclusion and its interaction with other materials [66]. This carefully designed composition resulted in the material showing a distinctive combination of magnetic and electrical responses, increasing its potential for use across various industries. Notably, the composite material has been shown to be exceptionally well suited for use in magnetic actuators with self-sensing strain characteristics, owing to its outstanding response time and remarkable reproducibility. This was also observed when PVDF fibers were incorporated with α-Fe_2_O_3_ nanoparticles owing to the enhancement of the crystallization of PVDF that eventually resulted in the polarization of PVDF nanofibers and the subsequent formation of an electroactive β-phase. Notably, the composite material has been shown to be exceptionally well suited for use in magnetic actuators with self-sensing strain characteristics, with an outstanding response time and remarkable reproducibility [67].

### 3.5. pH- and Ion-Sensing Polymer Actuators

The chemical environment in which reactions occur is important for controlling and manipulating the outcome of the reactions. Hence, adjusting the pH can be useful. Occasionally, photochemical copolymers are obtained by combining methylene-bis-acrylamide with acrylamide. The development of pH sensors on polymer gels has drawn considerable attention. The operation principle of such a polymer gel-based sensor depends on its swelling value, which increases or decreases depending on the pH value. Furthermore, stress variations have an impact and generate variant electrical impulses that can be recorded and interpreted. Advances in pH sensors have been achieved through miniaturization techniques, and thin films of poly(2-hydroxyethyl methacrylate), 2-di-methylamino ethyl methacrylate, etc., with thicknesses of around 10 µm exhibit a remarkable capacity for dissociation, heightened sensitivity to hydronium ions, and high measurement accuracy for the hydronium ion concentration in their immediate environment. Additionally, CPs such as poly(p-phenylenediamine) have shown potential for use as sensors in electronic applications. In CPs, delocalized electrons are present in the π-bonds in the aromatic rings along with the double bonds within the carbon chains.

**Figure 10:**
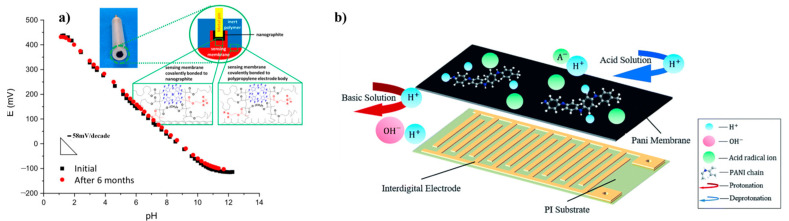
(**a**) Initial emf response (squares) and response after 6 months (circles) to pH for an ISE with a PDMA membrane (doped with ionophore and ionic sites) photographed onto a polypropylene-based electrode body and nano-graphite solid contact, relative to a free-flowing double-junction reference electrode. The pH was adjusted by the addition of 1.0 M HCl or 1.0 M NaOH to 10 mM sodium phosphate buffer solution (pH 7.1). The pH shown on the x-axis was measured using a pH glass electrode.Reprinted with permission from reference [68]. (**b**) schematic representation of our pH sensor consisting of a PANI membrane on interdigital electrodes supported by a PI substrate. The transformation of PANI protonated in acid solution and deprotonated in basic solution [69].

In photoinduced graft polymerization, specialized ionophore-doped crosslinked poly(decyl methacrylate) sensing membranes are intricately linked to high-surface-area carbon and inert polymeric electrode body materials. This intricate link facilitates the precise and sensitive detection in various applications. Examples of these materials are polypropylene and poly(ethylene-co-tetrafluoroethylene). These pH sensors, which have a covalently attached H+ selective ionophore, offer advantages over traditional pH glass electrodes (see Figure 10a) [47]. This method is suitable for fabricating adaptable ion-selective electrodes capable of detecting various substances. A conductimetric-type micro pH sensor was fabricated on a flexible film substrate in a study by using a PANI membrane. This sensor had a PANI membrane, an interdigital electrode, and a polyimide as the substrate, as depicted in Figure 10b [48]. When PANI was doped with dodecylbenzene sulfonic acid, it exhibited enhanced conductivity. Furthermore, it was found that the material showed a sensitivity of 58.57 mV/pH across the entire range of the pH spectrum (i.e., from 5.45 to 8.62).

### 3.6. Gas-Sensing Polymer Actuators

**Figure 11:**
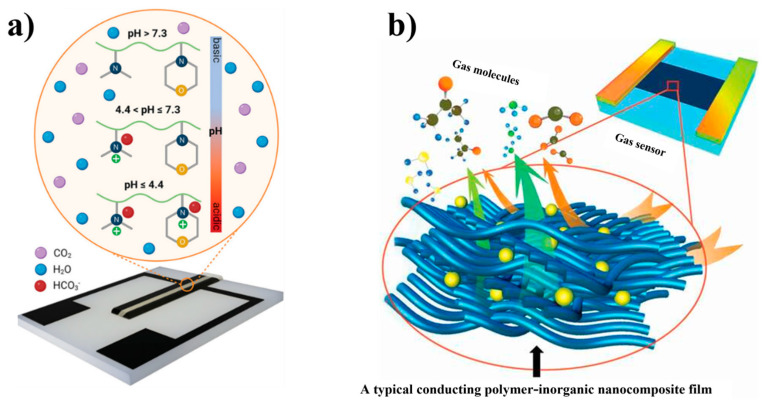
(**a**) The schematics of the fabricated p(D-*co*-M) sensor and the copolymer’s predominant protonation state at various pH values. Reprinted with permission from reference [72]. (**b**) A schematic diagram representing the deposition of gas molecules on the surface of a conducting polymer composite film consisting of inorganic nanoparticles. Reprinted with permission from reference [73].

Polymer-based gas sensors can detect the presence of target gases by measuring electrical changes within the polymer. These changes are induced either by redox reactions between target gas molecules and functional groups present in the doping agent or monomer or by charge transfer processes within the polymer [70,71]. Recently, remarkable progress has been made in fabricating gas sensors by using polymer–inorganic nanocomposites. Figure 11a [72] demonstrates this research, and the text discusses how inorganic nanomaterials play an important role in enhancing the gas-sensing capabilities in conducting polymers. It specifically highlights the effectiveness of a copolymer—poly(N-[3-(dimethylamino)propyl]-methacrylamide-co-2-N-morpholinoethyl methacrylate) (p(D-co-M))—in detecting a wide range of CO_2_ concentrations, as depicted in Figure 11a. This specific type of polymer has been used to develop a chemoresistive, affordable, flexible, and reversible CO_2_ sensor. It is noteworthy that sensors with p(D-co-M) show outstanding selectivity for CO_2_, even in the presence of interfering gases such as methanol, ethanol, toluene, and acetone.

Conductive polymers, such as PVC and PMMA, and their composites have been used to develop gas sensor devices. These polymers, along with other polymers with similar characteristics, play an important role in the functionality of the sensor devices (refer to Figure 11b) [73]. The polymers possess active functional groups in their structure that strongly influence the gas sensor performance. The ability of gas sensors to detect atmospheric moisture or humidity makes them highly applicable in industries and biomedical applications. In sensors used for humidity measurements, polymers such as poly(vinyl alcohol) are preferred, as they possess hydrophilic properties that affect their conductive properties depending on the amount of moisture absorbed. However, certain polymers with functional groups such as -SO_3_H, -COOH, and -N+(R)3Cl show low stability in the presence of moisture owing to their water solubility. Hence, specific nanomaterials or intermediates should be added to obtain hydrophilic polymers that exhibit high stability in the presence of moisture.

### 3.7. Stress-Sensing Polymer Actuators

**Figure 12:**
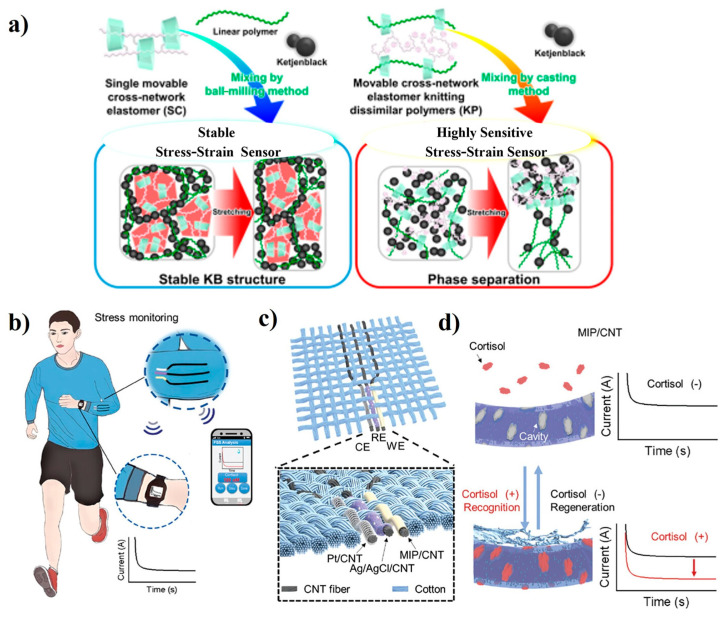
Proposes structures of (**a**) SC/PEA/KB(10) and (**b**) PSCD⊃PEA/KB(10). Reprinted with permission from reference [74]. Schematic illustration of the wearable FSS for real-time stress management. (**b**) Schematic illustration of the wearable FSS that enables cortisol monitoring through a CNT-based sensor with an MIP. (**c**) Schematic illustration of a single fabric sensor and magnified image of the fabric sensor. (**d**) Schematic illustration and current response in cortisol recognition. The red solid line indicates the current response after cortisol recognition. Reprinted with permission from reference [75].

Stress-sensing polymers measure stress by detecting changes in electrical conductivity, which occur when external strain is applied. These changes arise from the movement of internal defects or the formation of cracks, which induce the tunneling effect within the polymer [16]. The potential use of stress sensing polymer actuators in soft robotics and biomedical engineering is a fascinating area of research. These smart polymers, with photoluminescence properties that change with deformation or other forces, open up a world of possibilities for applications in stretching or shearing. Elastomer nanocomposites engineered with flexible crosslinks and incorporated ketjenblack, a type of carbon filler, have been developed. These composites exhibit exciting behavior, as their electrical resistance increases in direct proportion to the tensile strain, which indicates their potential for use as stress–strain sensors (refer to Figure 12a) [74]. Notably, these composites show high sensitivity as stress–strain sensors, and the exponential increase in their resistance as the tensile strain increases is attributed to the dethreading of the polymer from CD rings. Furthermore, an innovative fabric sensing system with a fiber electrode has been developed for rapid and accurate cortisol detection. This advanced system has exceptional repeatability, breathability, and durability. The fibers are composed of aligned carbon nanotubes and have been functionalized with a molecularly imprinted polymer that contains redox-active nanoreporters (refer to Figure 12b) [75]. This cutting-edge fabric sensing system facilitates effortless and continuous monitoring of cortisol levels, providing invaluable insights into stress levels, emotions, and overall health conditions.

## 4. Fabrication Techniques

Recent advances in polymer fabrication techniques have led to the development of various polymer sensors and actuators with a wide range of applications in soft robotics, especially in biomedical engineering. The recent development of electroactive polymer actuators with significantly improved intrinsic properties, such as the ability to respond to electrical stimuli and generate mechanical motion, has played a pivotal role in various applications [76]. These include robotics, medical devices, and artificial muscles. Moreover, the potential of 3D printing in the fabrication of advanced actuators has opened new possibilities for developing complex and precisely designed actuators tailored to specific applications. Through 3D printing, it is now possible to fabricate actuators with intricate geometries and structures that can respond to different types of stimuli. These recent developments in polymer fabrication have provided new opportunities for constructing advanced soft robotics systems that can be used in various biomedical applications, such as prosthetics, surgical devices, and medical implants, which has instilled a sense of optimism and hope for the future.

### 4.1. Electrochemical Method

**Figure 13:**
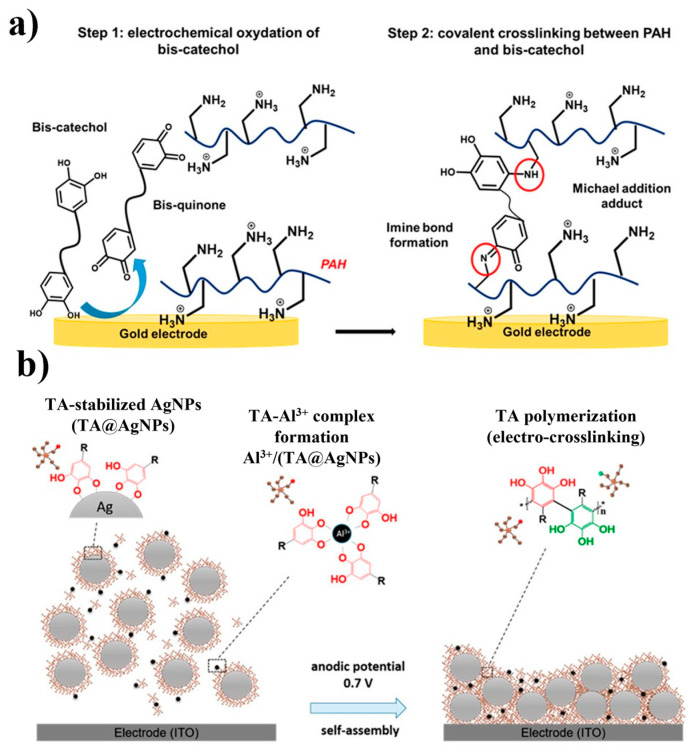
Electrosynthesis strategies are based on the direct formation of quinones. Schematic representations of the electro-crosslinking, through direct electro-oxidation of catechol/gallon moieties of (**a**) PAH/bis catechol film [77]. (**b**) Representation of Al^3+^/TA@AgNP film assembly. Reproduced with permission from reference [78].

The process of electrochemically triggered self-assembly of films presents a straightforward and effective method for depositing compounds with outstanding functionalities onto microelectrode arrays, as illustrated in Figure 13a [77]. Electrochemical polymerization offers several advantages, including high thermal stability, efficient redox reactions, and slow degradation rates. However, it also has drawbacks: polymer chains may undergo hydrolysis or over-oxidation, leading to a decrease in the electroactivity and conductivity of the conductive polymer. The self-assembly of films by using catechol is an interesting example. In this process, ethylene glycol molecules containing catechol groups at both ends, called bis-catechol molecules, are combined with commercially available poly(allylamine hydrochloride) (PAH) chains in an aqueous solution. Subsequently, this mixture is exposed to an electrode for further processing. When subjected to specific potential cycles, the bis-catechol molecules undergo oxidation, which results in the formation of highly reactive quinone groups. These groups undergo specific chemical reactions with the amino groups of PAH, ultimately forming a thin sensor film that can be used for the electrochemical tracing of aluminum ions.

The methodology involves the use of tannic acid (TA) and silver nanoparticles (AgNPs), which are ionically imprinted through electrodeposition, as depicted in Figure 13b [78]. In this procedure, the printing parameters are meticulously fine-tuned for depositing TA films onto an indium tin oxide (ITO) electrode. This is accomplished using aluminum ions through electrodeposition. Later, AgNPs are synthesized and combined with TA and aluminum ions to form a film on the ITO surface. Thus, this method produces a mesh-structured layer of AgNPs interlinked with TA through covalent bonds.

### 4.2. Self-Assembly Method

**Figure 14:**
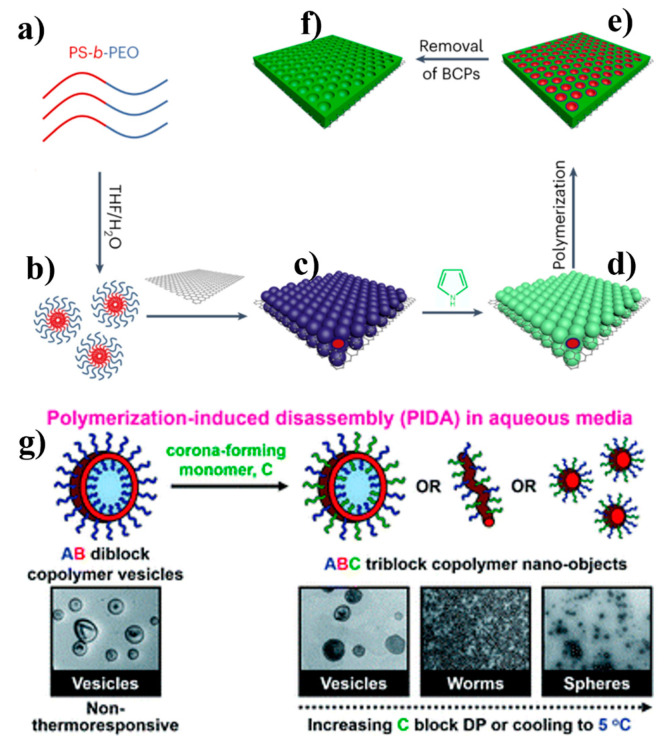
(**a**) Schematic illustration of constructing a mesoporous CP layer on 2D functionalized surfaces. (**a**) Simplified schematic diagram of PS-b-PEO. (**b**) PS-b-PEO dissolved in the mixed solution to form spherical micelles. (**c**) The micelles are tightly arranged on the GO surface. (**d**) Micelles attract Py monomers to form complex micelles. (**e**) Monomer polymerizes in situ to form a polymer network. (**f**) Removal of the template to obtain sandwich-structured mesoporous PPy nanosheets [80]. (**g**) Schematic illustration of the synthesis route employed for the preparation via RAFT-mediated aqueous polymerization-induced disassembly (PIDA). Reproduced with permission from reference [81].

A novel and robust technique has been devised to produce a variety of 2D mesoporous conducting polymer nanosheets [79]. These nanosheets exhibit customizable pore sizes ranging from 5 to 20 nm and thicknesses spanning 13 to 45 nm. Their shape and composition can be tailored through solution-based self-assembly (see Figure 14a) [80]. The preparation of a 2D mesoporous conducting polymer typically takes 12 h. Subsequently, supercapacitor testing takes roughly 24 h, and the testing of Na-ion batteries takes around 72 h. This entire process is best suited for individuals with expertise in physics, chemistry, materials science, and related fields. In a recent study, researchers used G-H vesicles as the initial seeds for the reversible addition–fragmentation chain transfer (RAFT) aqueous dispersion polymerization of oligo(ethylene glycol) methyl ether methacrylate, as illustrated in Figure 14b [81]. The process of polymerization-induced disassembly transformed the original precursor vesicles into lower-order worm or spherical structures. This transformation was induced for achieving the desired mean degree of polymerization for the corona-forming POEGMA block. This innovative method can potentially simplify the systematic design of new nanostructures by facilitating the achievement of a customizable composition, copolymer architectures, and properties that respond to various stimuli. Self-assembly polymerization has the advantage of being easy and simple. However, it is also characterized by lower stability and the tendency to form aggregates, which are significant drawbacks [82].

### 4.3. Dispersion Techniques

Dispersion polymerization is a technique used to produce polymers in the form of colloids or fine particles (200 nm to a few micrometers), and it is widely used in applications such as the preparation of coatings, in bioassays, in the manufacture of printing inks, in separation processes, and in the fabrication of catalyst supports [58]. It is a complex process involving two key techniques. Reversible deactivation radical polymerization (RDRP) and RAFT polymerization are two advanced polymerization techniques that facilitate precise control over the structure and properties of polymers. The advantage of the radical polymerization process lies in the continuous progression and maintenance of the polymerization process, which is based on the stability of the control agent and the independence of interactions. However, undesired reactions occurring during polymerization can lead to the loss of the control agent, making it difficult to obtain polymers with the desired molecular weight [82,83,84].

#### 4.3.1. Reversible Deactivation Radical Polymerization (RDRP)

RDRP involves two main methods: atom transfer radical polymerization (ATRP) and nitroxide-mediated polymerization (NMP). In ATRP, metal catalysts are used to facilitate the atom transfer radical addition reaction of alkyl halides and alkenes to form 1:1 adducts. This method includes a reversible redox process catalyzed by a transition metal complex, which generates radicals that combine with intermediate radicals to form polymer chains. The polymerization process can be terminated through radical coupling or disproportionation [85]. NMP is controlled using nitroxide radicals, leveraging a reversible transition mechanism between growing propagating radicals and nitroxide radicals to obtain alkoxyamines [11]. Pan et al. synthesized a dumbbell-shaped block copolymer for the fabrication of soft actuators by crosslinking PDMS side chains with poly(butyl acrylate) using the RDRP method. They used a PFMA/PAMA random copolymer, formed via the random copolymerization of furfuryl methacrylate and post-crosslinkable allyl methacrylate (AMA), as a macroinitiator to synthesize poly(dimethylsiloxane methacrylate) and AMA diblock and triblock copolymers. The use of various initiators in the RDRP method helped to successfully produce the dumbbell-shaped triblock copolymer. The side chain length of the resulting triblock copolymer was approximately 9.3 nm, corresponding to a maximum diameter of about 18.6 nm. A star-shaped gripper fabricated from this dumbbell-shaped triblock copolymer could be bent up to 95° in a tetrahydrofuran solution, while the orthogonal bilayer could be bent up to 330°. The advantage of the RDRP process lies in the continuous progression and maintenance of the polymerization process, which is based on the stability of the control agent and the independence of interactions. However, undesired reactions occurring during polymerization can lead to the loss of the control agent, making it difficult to obtain polymers with the desired molecular weight [86,87].

#### 4.3.2. Reversible Addition–Fragmentation Chain Transfer (RAFT) Polymerization

RAFT allows for more precise control over the polymerization process, making it the most widely used method to date [88]. Researchers suggest that this method allows for the production of high-density anisotropic toroidal polymers, which can be utilized to fabricate nanoparticles and nanoactuators [86].

**Figure 15:**
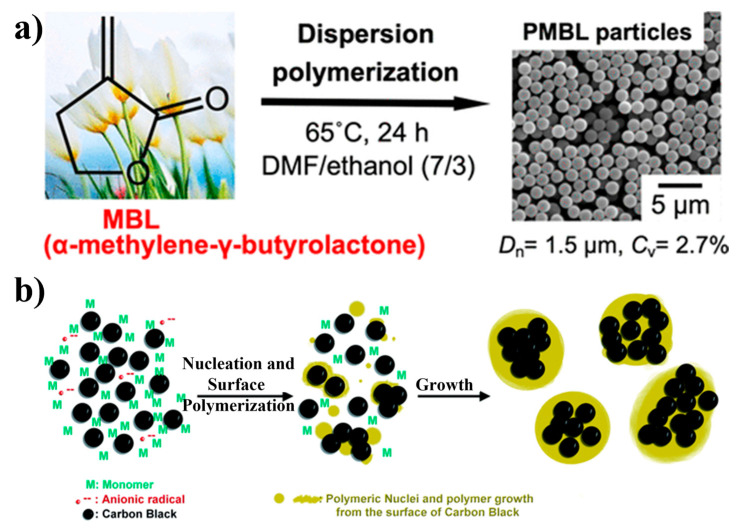
(**a**) Dispersion polymerization of tulip-derived α-methylene γ-butylrolactone (MBL). Reproduced with permission from reference [89]. (**b**) Schematic illustrating the Au assembly process of polymer substrates. Reproduced with permission from reference [90].

At a temperature of 65 °C, the researchers produced uniformly sized bio-based polymer particles. This was accomplished by dispersing tulip-derived α-methylene-γ-butyrolactone (MBL) in a mixture of N, N-dimethylformamide and ethanol (7:3, *w*/*w*). To maintain uniformity, they employed poly(vinylpyrrolidone) (PVP) as a colloidal stabilizer, as illustrated in Figure 15a [89]. The researchers achieved the ability to precisely regulate the size of the polymer particles by adjusting the composition of the reaction medium or varying the concentration of PVP. Furthermore, an innovative technique was introduced that utilizes surfactant-free aqueous/alcoholic dispersion polymerization to encapsulate nanoparticles within polymer latex, as depicted in Figure 15b [90]. The researchers employed a novel method to develop a composite latex consisting of polystyrene (PS) and carbon black (CB). They modified the surface of the CB by introducing reactive functional groups, which enabled it to participate actively in nucleation and surface polymerization. This led to the formation of a well-defined structure in which the CB was encapsulated within the PS composite latex. This also allowed for the control of the thickness of the polymer shell by adjusting the weight ratio between the carbon black and the monomer, allowing for the tailored and precise fabrication of the composite structure.

### 4.4. 3D Printing

Three-dimensional (3D) printing technology, developed in 1980, facilitates the production of computer-modeled objects through the successive layering of materials. The advantages of 3D printing polymerization include the ability to obtain desired shapes through 3D modeling, ease of fabrication, and minimal material waste. However, limitations such as the restricted range of printable materials, potential issues with printing quality, and challenges in large-scale production are notable disadvantages [89]. When a three-dimensional structure based on computer-aided design (CAD) is input into a 3D printer, the printer constructs the object by depositing material layer by layer according to the specified structure. Owing to the high degree of freedom of both material and structure, this technology is widely used across academia and various industries, including to produce artificial joints [65], rocket engines [66], and shoes [66,67]. Recently, advances in 3D printing technology have made it possible to print polymer composites directly. Major techniques include stereolithography (SLA), which involves a laser to cure photopolymer resins layer by layer, and inkjet printing, which deposits liquid polymers in a similar layer-by-layer fashion. The applicability of 3D printing with a wide range of materials and structures makes it highly useful across diverse industrial sectors [68]. Yuk et al. fabricated a unique 3D printable conducting polymer ink derived from PEDOT:PSS. This innovative ink has distinctive properties that make it well suited for various applications. The prepared PEDOT:PSS ink could be deposited through a 100 μm nozzle, facilitating the fabrication of structures up to 20 layers thick, and it could create protruding shapes. The ink could facilitate the printing of over 100 circuits, each being 100 μm in size, within 30 min, facilitating the production of highly flexible circuits without mechanical failure [69]. Dong et al. employed a unique approach, blending digital light processing (DLP) with polymerization-induced phase separation techniques to craft an intricate, porous, macroscopic 3D polymer structure. DLP is an additive manufacturing technique that employs projected light patterns to precisely polymerize printed ink, and it can facilitate the construction of three-dimensional objects. The 3D object was constructed layer by layer from printing ink, with local photopolymerization induced by patterned UV light. This process led to phase separation between the polymer-rich and polymer-poor phases owing to the thickness of the polymer ink. Subsequently, the printed 3D object was subjected to a process of monomer and porogen removal by soaking it in acetone for 24 h. The researchers successfully controlled the porosity of the 3D object by adjusting the concentrations of the porogens, 1-decanol, and cyclohexanol. This allowed them to precisely manipulate the nanopore sizes, ranging from 10 nm to 1000 μm, in the object. The researchers proposed that these nanoporous polymer objects could be used in various fields such as the fabrication of 3D cell culture scaffolds and in filtration and catalysis [70].

**Figure 16:**
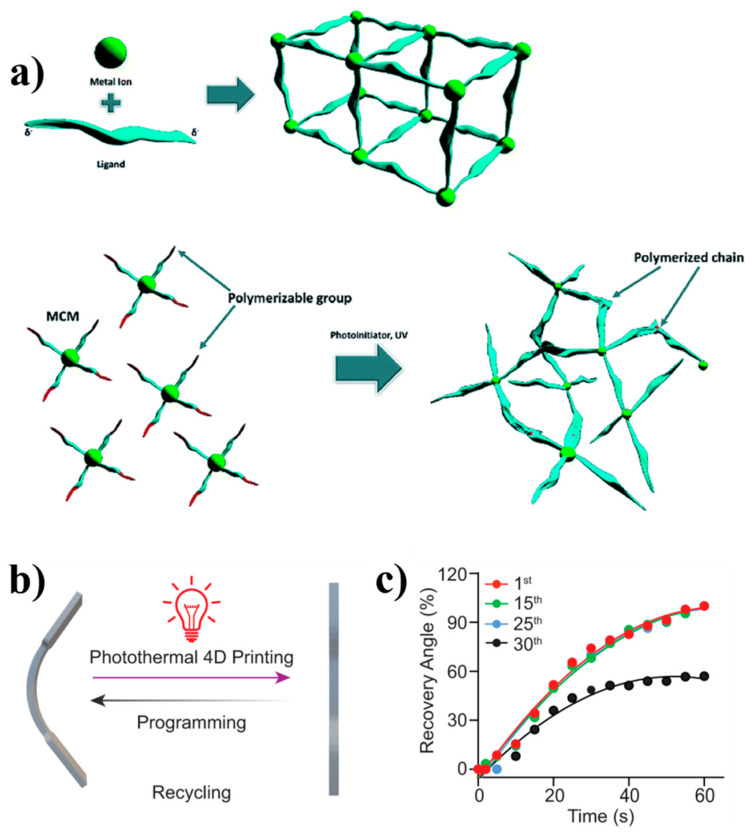
(**a**) CP formation by reacting metal ions with bridging ligands and also CP formation via polymerization of MCMs. Reproduced with permission from reference [97]. (**b**) The recycling process of shape-memory polymers in repeated programming and photoinduced 4D printing. (**c**) The recovery angle versus time of LMPCs in a total of 25 cycles while irradiating with 808 nm laser (0.3 W/cm^2^) for 60 s. Reproduced with permission from reference [98].

Researchers have made significant strides in creating 3D free-standing objects using CP materials. They have employed coordination metal complexes as the fundamental building blocks and a 3D printer to synthesize a coordination polymer and shape it into a 3D object concurrently. Using a direct print-and-form approach, they have created a 3D-shaped nickel tetraacrylamide monomeric complex without using a binder. This innovative process shows the effectiveness of the use of CPs in producing complex structures [71]. In one study, researchers employed state-of-the-art reversible addition–fragmentation chain transfer (RAFT) polymerization agents attached to liquid metal nanoparticles. This pioneering approach led to a significant improvement in light-mediated near-infrared 4D printing. The innovative method facilitated the photothermal-induced 4D printing of composites, resulting in the rapid restoration of their original shape within just 60 s when exposed to light (refer to Figure 16b). These findings provide a glimpse into the exciting potential use of liquid metal–polymer composites in the realm of 4D printing, underscoring their remarkable promise in the field of soft robotics [72].

Kulkarni et al. synthesized a polymer actuator capable of actuation through thermal expansion by using a 4D printing technique via SLA. They prepared a composite of spin crossover (SCO) monomers [Fe(NH2trz)_3_]SO_4_ and Fe(NH_2_trz)_3_(SiF_6_)0.5 with photocurable resins DS3000 and PEGDA-250. SCO is a phenomenon where the spin state within a material alternates between high-spin and low-spin states in response to external stimuli, such as temperature changes. The thermal energy applied during SCO is converted into kinetic energy, which induces actuation. Differential scanning calorimetry analysis of the SCO properties of the polymer complex fabricated via SLA printing showed peaks around 72 °C and 56 °C for [Fe(NH_2_trz)_3_]SO_4_@DS3000, corresponding to SCO. For actuation analysis, a bilayer stick structure with dimensions of 5 mm (length), 0.5 mm (width), and 0.045 mm (thickness) was fabricated. The active layer was composed of [Fe(NH_2_trz)_3_]SO_4_@PEGDA_30, while the inactive layer was made of PEGDA-250 containing 66 wt% BaTiO_3_ particles. An experiment showed that when the temperature was increased from 25 °C to 90 °C, approximately 3 mm of bending occurred, with a reactive force of about 0.15 mN [91].

As the range of materials suitable for 3D printing continues to expand and with the advancement of 4D printing and increased technological precision, it is expected that polymerization via 3D printing will find even broader applications in various fields such as medicine, biotechnology, superhydrophobic objects, and soft robotics [92].

### 4.5. 4D Printing

Four-dimensional (4D) printing is considered a very innovative technology, and it involves the use of additive manufacturing, SMPs, and 3D printing. The resulting structures are of high value, for they can change their shape with time [93,94,95]. Hydrogels are highly beneficial in 4D printing owing to their biocompatibility with cells, organs, and living tissues. This technology is expected to be widely used in soft robotics, as it can respond to external stimuli such as moisture, light (refer to Figure 16b), magnetism, and electricity. The materials fabricated using 4D printing can function independently to maintain quality performance and time precision. This technology is widely used in the field of biomedical science for drug delivery, in dentistry, and biomedical devices [96]. However, it also finds versatile applications in soft robotics, construction, agriculture, and electronics.

## 5. Conclusions and Future Perspectives

Polymer sensors have been an important component of soft robotics for many years. However, recently, our understanding of their behavior has improved, which has allowed us to exploit their potential fully. Polymer sensors have been fabricated using various methods and used for many purposes, some of which are highlighted here. Soon, the synergistic combination of stimuli-responsive polymers and nanoscale materials is poised to revolutionize various industries owing to their novel functions and properties. To support this progress, researchers should develop theories to explain these newfound behaviors, which would facilitate the smart design of polymers with new materials tailored to specific applications. Another challenge is the development of systems that can intelligently and predictably respond to multiple external stimuli. These materials are important for accelerating the development of biomimetic systems with high stability and longevity. Efforts to exploit their sensitivity to external stimuli and integrate them with different technology areas should be intensified. Polymer actuators can be used as sensors in novel ways in soft robotics, and they can operate accurately and reliably alongside the latest engineering wearables to provide integrated monitoring functionalities. This review covers different types of polymers and their use as stimuli-responsive smart sensors in soft robotics.

## Data Availability

Data sharing is not applicable to this article.
